# Editorial: Insights in glomerular disease

**DOI:** 10.3389/fneph.2024.1480968

**Published:** 2024-12-12

**Authors:** Bryan Chang, Abbal Koirala, Duvuru Geetha

**Affiliations:** ^1^ Department of Medicine, University of Cambridge, Cambridge, United Kingdom; ^2^ Division of Nephrology, Department of Medicine, Johns Hopkins University School of Medicine, Baltimore, MD, United States

**Keywords:** glomerulonephritis, membranous nephropathy, lupus nephritis, Bartonella infection, pregnancy

Glomerulonephritides vary in presentation and can cause fluid overload, hypertension, and kidney failure. They are either primary (intrinsic to the kidney), such as minimal change disease or membranous nephropathy, or secondary (part of systemic disease), such as vasculitis, lupus nephritis, or infection-associated glomerulonephritis.

Over the past century, scientists have made progress in elucidating the pathogenesis of glomerular diseases and their clinical presentations, for instance, through the discovery of anti-nephrin antibodies (1, 2) in adult and anti-UCHL1 (3) and anti-annexin 1 antibodies (4) in pediatric minimal change disease, or discovery of anti-PLA2R antibodies (5) in idiopathic membranous nephropathy. However, the pathology, serological profiles, disease presentations, and specific management of atypical etiologies or physiological states, e.g., pregnancy on glomerulonephritides, are less well characterized. This is partly due to the lack of data, which comprises mainly of case series/reports, and the only recent discovery of proteins such as neural epidermal growth factor-like 1 (NELL1) in the case of membranous nephropathy. In this Research Topic, three articles focusing on an NELL1-associated membranous nephropathy (MN), the rare blood-culture negative Bartonella infective endocarditis (IE)-associated glomerulonephritis and an overview of lupus nephritis in pregnancy are presented. They highlight the diagnostic challenges and the need for informed and specific management relevant to these disease states.

Andeen et al. summarize the existing literature on NELL1-associated MN. MN was historically classified as idiopathic (associated with anti-PLA2R antibodies) or secondary to underlying malignancy, autoimmune disease, or infection (6). However, the recent use of laser microdissection and mass spectrometry in PLA2R-negative MN has revealed the existence of new antigens, such as NELL1. Interestingly, NELL1 MN can be idiopathic, associated with autoimmunity and malignancy, as well as thiol-containing medications, e.g., lipoic acid or traditional indigenous medications (TIM). They highlight a possible epidemiological variation in pathogenesis, with the majority of patients in India taking TIM, while malignancy is more reported in Western countries such as the US. The differences in etiology may be clinically significant due to the higher remission rates for NELL1 MN on TIM compared to other associations, with implications for more conservative management rather than initiating immunosuppressants for the former cases. They also highlight 7 patients with dual PLA2R and NELL1 positivity and suggest that in patients with weak PLA2R expression, this may be a false positive tissue reaction and warrant testing for NELL1.

It remains unclear whether the NELL1 antigen plays a causal role in MN since its function in the kidney is unknown. However, its overexpression has been linked with increased osteoblast differentiation and craniosynostosis (7). It may be a cryptic antigen expressed following an insult from medication or malignancy (6). The mechanisms behind its expression are also unclear, although the authors suggest that disruption of disulfide bonds in thiol-exposure cases may result in the exposure of neo-epitopes (8), such as NELL1. TIM, particularly fairness creams in India, contain high levels of mercury, which has a high affinity for sulfanyl groups on the GBM and acts as a hapten, eliciting antibodies targeting antigens, including possibly NELL-1, leading to immune complex deposition (9). However, given its other associations, including malignancy, further research is needed to untangle the mechanisms behind these associations.

Kitamura et al. provide a pooled analysis of Bartonella IE-associated glomerulonephritis from case reports/series. It is a leading cause of culture-negative IE, and they highlight important clinicopathological differences from culture-positive IE-associated glomerulonephritis. Salient differences of Bartonella IE-associated glomerulonephritis include delayed onset to kidney biopsy, frequency of serological markers of inflammation, including ANCA (75%) and RF positivity (84%) and low C3/C4 levels, and a higher prevalence of crescents and necrotizing lesions and immune deposits on biopsy (IgG, IgM>IgA, C3 and C1q). Additionally, due to incomplete data from the literature, they present four in-house cases that provide an overview of the clinical and serological markers found.

The presence of such autoimmune markers, its fastidious nature, combined with non-specific malaise, fever, purpuric rashes, and renal dysfunction, poses a diagnostic challenge to clinicians that may favor other diagnoses such as ANCA-vasculitis or lupus nephritis, which is suggested by at least 45 cases treated with glucocorticoids. The consequences of missed or delayed diagnosis can be severe, and they highlight that 53% of the analyzed cases required valve replacement therapy comparable to the general rate in IE patients and illustrate its potential impact on cardiac function (10). A detailed history, including exposure to cats and a high index of suspicion, especially when faced with atypical autoimmune profiles inconsistent with ANCA-vasculitis and positive serological testing for Bartonella, is important so as not to miss this important diagnosis.

Lupus nephritis (LN) is a glomerulonephritis arising from Systemic Lupus Erythematosus, a chronic autoimmune disease in which women of childbearing age are particularly implicated. In pregnancy, the disease results in increased risk to maternal health, including pre-eclampsia, hypertension, and thromboembolic events, as well as fetal risks, such as preterm birth, congenital heart block, and intrauterine growth restriction (11). Therefore, management of pregnancy in lupus nephritis patients requires careful planning and thorough monitoring for possible complications. Ghozloujeh et al. review the current management approach to optimize care in such patients. They highlight the importance of good disease control before pregnancy, using a disease activity index score to assess activity since the incidence of flares is much higher for those with active LN (12), and contraceptive options for those with active disease should be discussed. During pregnancy, while hydroxychloroquine is a mainstay therapy to maintain remission (13), others, such as cyclophosphamide, are contraindicated or have limited safety data. For managing lupus nephritis flare, glucocorticoids, azathioprine, and calcineurin inhibitors are deemed acceptable in pregnancy (14). With the increasing advent of biologics, further research is required to understand their safety profiles. In the meantime, careful consideration of risks and benefits should be made through shared decision-making.

LN flares can be challenging to diagnose past 20 weeks gestation as they may be confused with pre-eclampsia, which features hypertension and proteinuria. The authors highlight distinguishing markers, including the sFLT-1/PIGF ratio, which is increased in pre-eclampsia compared to lupus nephritis flare (15); however, this biomarker is not routinely available in clinical use. Similarly, serum anti-NELL antibodies are not commercially widespread, highlighting the need for improved clinical translation from the ‘bench to bedside’ to enhance diagnostic precision in various glomerulonephritides.

## References

[1] Watts AJB Keller KH Lerner G Rosales I Collins AB Sekulic M. Discovery of autoantibodies targeting nephrin in minimal change disease supports a novel autoimmune etiology. J Am Soc Nephrol. (2022) 33:238–52. doi: 10.1681/ASN.2021060794 PMC8763186 34732507

[2] Hengel FE Dehde S Lassé M Zahner G Seifert L Schnarre A. Autoantibodies targeting nephrin in podocytopathies. N Engl J Med. (2024) 391:422–33. doi: 10.1056/NEJMoa2314471 38804512

[3] Jamin A Berthelot L Couderc A Chemouny JM Boedec E Dehoux L. Autoantibodies against podocytic UCHL1 are associated with idiopathic nephrotic syndrome relapses and induce proteinuria in mice. J Autoimmun. (2018) 89:149–61. doi: 10.1016/j.jaut.2017.12.014 29307588

[4] Ye Q Zhang Y Zhuang J Bi Y Xu H Shen Q. The important roles and molecular mechanisms of annexin A2 autoantibody in children with nephrotic syndrome. Ann Transl Med. (2021) 9:1452. doi: 10.21037/atm-21-3988 34734004 PMC8506724

[5] Beck LH Jr Bonegio RGB Lambeau G Beck DM Powell DW Cummins TD. M-type phospholipase A2 receptor as target antigen in idiopathic membranous nephropathy. N Engl J Med. (2009) 361:11–21. doi: 10.1056/NEJMoa0810457 19571279 PMC2762083

[6] Sethi S. The many faces of NELL1 MN. Clin Kidney J. (2023) 16:442–6. doi: 10.1093/ckj/sfac237 PMC9972807 36865014

[7] Ting K Vastardis H Mulliken JB Soo C Tieu A Do H. Human NELL-1 expressed in unilateral coronal synostosis. J Bone Miner Res. (2009) 14:80–9. doi: 10.1359/jbmr.1999.14.1.80 9893069

[8] Santoriello D Ramaswamy R Kudose S Markowitz GS. Segmental NELL-1 membranous nephropathy complicating tiopronin therapy. Kidney Int Rep. (2023) 8:1683–6. doi: 10.1016/j.ekir.2023.05.023 PMC10403669 37547528

[9] Narayanan R Sivadas S Kurien AA. NELL-1-associated membranous nephropathy linked to skin fairness cream use: insights from an Indian case series. Kidney Int. (2024) 105:1316–9. doi: 10.1016/j.kint.2024.03.025 38615743

[10] Cuervo G Escrihuela-Vidal F Gudiol C Carratalà J. Current challenges in the management of infective endocarditis. Front Med. (2021) 8:641243. doi: 10.3389/fmed.2021.641243 PMC7937698 33693021

[11] Tan B So PN Krishnan A Carriazo S Bahamonde JR Lamech TM. Approach to pregnancy in patients with lupus nephritis. Kidney Med. (2023) 5:100724. doi: 10.1016/j.xkme.2023.100724 37915962 PMC10616386

[12] Attia DH Mokbel A Haggag HM Naeem N. Pregnancy outcome in women with active and inactive lupus nephritis: A prospective cohort study. Lupus. (2019) 28:806–17. doi: 10.1177/0961203319846650 31084235

[13] Clowse MEB Magder L Witter F Petri M. Hydroxychloroquine in lupus pregnancy. Arthritis Rheum. (2006) 54:3640–7. doi: 10.1002/art.v54:11 17075810

[14] Fakhouri F Schwotzer N Cabiddu G Barratt J Legardeur H Garovic V. Glomerular diseases in pregnancy: pragmatic recommendations for clinical management. Kidney Int. (2023) 103:264–81. doi: 10.1016/j.kint.2022.10.029 36481180

[15] Leaños-Miranda A Graciela Nolasco-Leaños A Ismael Carrillo-Juárez R José Molina-Pérez C Janet Sillas-Pardo L Manuel Jiménez-Trejo L. Usefulness of the sFlt-1/PlGF (Soluble fms-Like Tyrosine Kinase-1/Placental Growth Factor) Ratio in Diagnosis or Misdiagnosis in Women With Clinical Diagnosis of Preeclampsia. Hypertension. (2020) 76:892–900. doi: 10.1161/HYPERTENSIONAHA.120.15552 32713272

